# Comparison of Tibial Nail Entry Point Location Among Infrapatellar, Suprapatellar, and Lateral Parapatellar Approaches Using Postoperative 3D-CT

**DOI:** 10.3390/life16010087

**Published:** 2026-01-07

**Authors:** Takahiko Ichikawa, Suguru Yokoo, Yukimasa Okada, Junya Kondo, Keiya Yamana, Chuji Terada

**Affiliations:** Department of Orthopaedic Surgery, Fukuyama City Hospital, Hiroshima 721-8511, Japan; takahiko394@gmail.com (T.I.);

**Keywords:** Tibial shaft fractures, entry corridor, infrapatellar nailing, suprapatellar nailing, lateral parapatellar nailing, semiextended position

## Abstract

**Background:** Tibial shaft fractures are frequently treated with intramedullary nailing; however, malalignment remains a concern, particularly in proximal metaphyseal fractures. The surgical approach influenced the nail entry point; however, the three-dimensional (3D) geometric characteristics of the entry point among different approaches remain unclear. **Methods:** This single-center retrospective study included 68 patients with acute tibial shaft fractures (AO/OTA type 42) treated with reamed and locked intramedullary nails from January 2014 to June 2024. The surgical techniques employed included lateral parapatellar (LPA, n = 31), infrapatellar (IPA, n = 27), and suprapatellar (SPA, n = 10) approaches. Postoperative computed tomography (CT) data were reconstructed into standardized 3D images. The mediolateral insertion ratio was calculated as the percentage distance from the lateral tibial plateau edge to the nail entry point relative to the plateau’s width in the coronal plane. The shortest distance from the tibial articular surface to the nail (*r*) was measured in the sagittal plane. The Kruskal–Wallis test and Dunn’s post hoc comparisons were used to analyze group differences. **Results:** Baseline patient and fracture characteristics did not significantly differ among the groups. The mediolateral insertion ratio significantly differed (*p* < 0.0001), with a more lateral entry for the LPA (44.0% [43.0–47.0]) than for the IPA (51.0% [49.0–53.0], post hoc *p* < 0.0001) and SPA (49.0% [47.0–51.3], post hoc *p* = 0.0034). Further, the sagittal distance r significantly differed (*p* < 0.0001), with a more distal entry for the LPA (14.8 [12.8–20.1] mm) than for the IPA (9.7 [7.0–11.8] mm, post hoc *p* < 0.0001) and SPA (10.5 [5.5–12.9] mm, post hoc *p* = 0.0008). No statistically significant difference was observed between the IPA and SPA. **Conclusions:** The LPA generates a significantly more lateral and distal tibial nail entry point than the IPA and SPA. No statistically significant differences were detected between the IPA and SPA in either plane. These 3D-CT findings may warrant attention during approach selection and guidewire placement, particularly for fractures extending into the proximal metaphysis.

## 1. Introduction

Tibial shaft fractures are among the most prevalent long bone injuries and are frequently treated with intramedullary nailing, which is considered the standard of care because it enables stable fixation with minimal soft tissue disruption and early mobilization [1,2,3]. However, malalignment remains a clinically relevant complication, particularly in fractures that involve the proximal or distal metaphysis [1,4,5,6]. An inappropriate nail entry point has been identified as a key factor that contributes to malalignment, as it affects the association between the nail and the mechanical axis of the tibia [7,8]. Therefore, selecting an optimal entry point is crucial for achieving and maintaining alignment throughout the procedure.

Several surgical approaches have been developed to access the tibial entry point. The conventional infrapatellar approach (IPA) in knee flexion is extensively employed and familiar to many surgeons; however, it makes alignment control challenging because of the changing tibia orientation with knee flexion, and the patellar tendon must be split or retracted [9,10]. To address concerns regarding anterior knee pain associated with patellar tendon splitting and the difficulty of controlling alignment with the tibia in flexion, the SPA using a semiextended position was introduced, enabling nail insertion through the patellofemoral joint while maintaining the limb closer to the functional weight-bearing axis [4,9,11,12]. However, this intraarticular trajectory raises concerns about potential patellofemoral cartilage damage. More recently, extra-articular techniques, including the LPA, have been proposed to avoid intra-articular violation and reduce anterior knee pain by inserting the nail lateral to the patellar tendon without entering the joint [1,3,10,13,14]. Recent comparative studies have investigated suprapatellar (SPA), IPA, and LPA or extra-articular approaches in terms of alignment, knee function, and pain. However, these investigations have primarily relied on clinical outcomes and two-dimensional (2D) radiographs rather than direct three-dimensional (3D) assessments of the entry point [15,16,17,18].

Previous clinical and radiographic studies have compared these approaches in terms of postoperative alignment, anterior knee pain, and complication rates [2,4,9,11,13]. Some reports have indicated that semiextended techniques may improve alignment in proximal fractures; however, others have focused primarily on pain and functional outcomes of the procedure. Moreover, most prior investigations have indirectly investigated the entry point using 2D radiographs, which are affected by projection errors and do not fully capture the 3D relationship between the nail and the tibial plateau [7,8]. Consequently, the actual mediolateral and proximal–distal positions of the nail entry point associated with different approaches remain insufficiently clarified, particularly when comparing LPA, IPA, and SPA within a single cohort.

Three-dimensional computed tomography (3D-CT) permits the standardized evaluation of the nail entry point in both the coronal and sagittal planes, thereby providing more accurate geometric information than plain radiographs [5,19]. A better understanding of how each approach impacts the entry point location may help surgeons select the most appropriate technique for specific fracture patterns, especially when the fracture line extends into the proximal diaphysis, where small deviations in the entry point translate into clinically relevant malalignment.

Therefore, this study aimed to compare the 3D entry point positions of tibial intramedullary nails among the LPA, IPA, and SPA using postoperative 3D-CT reconstructions. We hypothesized that the LPA would lead to a more lateral and distal entry point than the IPA and SPA and that these geometric characteristics may have implications for coronal alignment control, particularly in fractures that involve the proximal metaphysis.

## 2. Materials and Methods

### 2.1. Study Design and Ethical Considerations

In this single-center retrospective observational study, we screened all consecutive patients who underwent intramedullary nailing for tibial shaft fractures from January 2014 to June 2024 at Fukuyama City Hospital for eligibility criteria. The Institutional Review Board (IRB) of Fukuyama City Hospital approved the study protocol (approval no. 846; 29 November 2024). The IRB waived the requirement for written informed consent; instead, participants or their guardians were informed about the study via an opt-out notice posted on our hospital website and displayed in the hospital. All data were anonymized before analysis, and the study was conducted following the Declaration of Helsinki guidelines.

### 2.2. Patient Selection

All consecutive patients who underwent intramedullary nailing for acute tibial shaft fractures at our institution during the study period were screened for eligibility criteria. Inclusion criteria were (1) the presence of an acute tibial shaft fracture classified as AO/OTA type 42; fracture location (proximal/middle/distal third) was determined according to the AO/OTA classification for the tibial diaphyseal segment [20]; (2) surgical treatment with intramedullary nailing performed at our institution; and (3) availability of postoperative CT images. Exclusion criteria were (1) pathological, periprosthetic, or refractures; (2) incomplete imaging data; and (3) additional plate fixation performed in combination with intramedullary nailing. At our institution, postoperative CT is routinely performed following tibial intramedullary nailing; however, in a small proportion of cases, CT was not obtained because of clinical considerations and/or practical constraints. Accordingly, the final analysis included 68 patients who met the inclusion criteria.

### 2.3. Surgical Approaches

The attending surgeon identified the surgical approach based on the fracture pattern and intraoperative considerations. LPA is defined as a skin incision lateral to the patellar tendon, enabling extra-articular insertion of the nail. The knee was positioned in a semiextended position. Direct intraarticular exposure was avoided in all cases. IPA is defined as a midline longitudinal incision over the patellar tendon with the knee flexed, and the nail was introduced using a patellar tendon–splitting approach. SPA is defined as a longitudinal SPA incision, and a specialized trocar system was inserted through the patellofemoral joint with the knee maintained in a semiextended position. Of the 68 patients, 31, 27, and 10 underwent LPA, IPA, and SPA, respectively. The distribution of surgical approaches over time is summarized in Table S1.

### 2.4. Implants

Reamed and locked intramedullary tibial nails were used for all fractures. Six commercially available tibial nail systems from the following four manufacturers were utilized: the Expert Tibial Nail and Tibial Nailing System (DePuy Synthes, Raynham, MA, USA), T2 Tibial Nailing System (Stryker, Kalamazoo, MI, USA), Natural Nail Tibial System and Phoenix Tibial Nail (Zimmer Biomet, Warsaw, IN, USA), and TRIGEN Meta-Nail tibial system (Smith & Nephew, Memphis, TN, USA). All implants had similar proximal bends and entry-point designs. The distribution of implant systems by surgical approach is summarized in Table S2.

### 2.5. Imaging Protocol and Reconstruction

**Figure 1:**
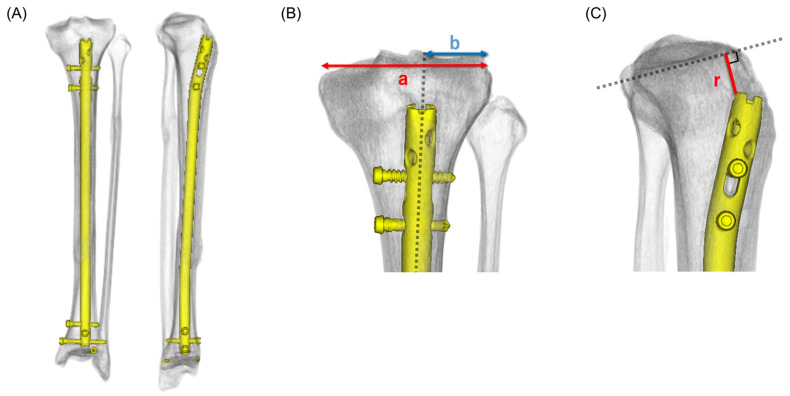
Postoperative CT data reconstructed into 3D images. (**A**) Images were reconstructed using the SYNAPSE VINCENT workstation (Fujifilm, Tokyo, Japan). (**B**) Frontal 3D reconstructed image. The transverse diameter of the tibial articular surface was defined as *a*, and the distance from the lateral edge of the articular surface to the intramedullary nail insertion point as *b*. The red arrow indicates *a*, and the blue arrow indicates *b*. (**C**) Lateral 3D reconstructed image. The shortest distance (*r*) from the tibial articular surface to the intramedullary nail was measured along a perpendicular line drawn from the articular surface. The red line indicates *r*.

Postoperative CT scans (slice thickness, 1 mm) were routinely obtained after surgery and were available for all included cases. CT data were reconstructed into 3D images utilizing a SYNAPSE VINCENT workstation (Fujifilm, Tokyo, Japan) (Figure 1A). The workstation’s bone extraction tools were used to segment the tibia and intramedullary nail, followed by manual refinement when necessary to remove non-target structures and reduce metal-related artifacts.

To standardize coronal and sagittal measurements, a tibial coordinate system was first established using the scanned tibial length (Ltibia_scan), measured as the cranio–caudal distance between the axial slice containing the tibial plateau articular surface (proximal reference) and that containing the tibial plafond (distal reference). Two axial cross-sections at 30% and 70% of Ltibia_scan (from the proximal reference) were subsequently selected. On each cross-section, the centroid of the tibial shaft was identified based on the cortical outline, and the line connecting the two centroids was defined as the tibial longitudinal axis (*Z*-axis).

The 3D volume was then reoriented by aligning the *Z*-axis with the vertical axis to minimize overall tilt. Axial rotation was adjusted by rotating the model around the *Z*-axis until a true lateral tibial profile was obtained, defined as minimal rotational asymmetry of the posterior cortical contours. Based on this orientation, the sagittal plane was defined by the true lateral orientation, and the coronal plane was defined as the orthogonal plane containing the *Z*-axis.

The mediolateral direction of the tibial plateau was defined for coronal-plane measurements using the most medial and lateral margins of the tibial plateau articular surface on the reconstructed model. This process enables consistent measurement of the transverse plateau width (a) and the mediolateral distance from the lateral plateau edge to the nail entry point (b). Reorientation was validated by confirming (i) an approximately level plateau appearance on the coronal view and (ii) a true lateral appearance on the sagittal view. The standardized coronal and sagittal views were subsequently utilized for quantitative measurements.

### 2.6. Measurement Parameters

Two measurements were performed. (1) Coronal plane analysis: The transverse diameter of the tibial plateau was defined as a. The distance from the lateral edge of the plateau to the center of the nail insertion point is defined as b. The mediolateral insertion ratio was calculated as (b/a) × 100 (%) (Figure 1B). A lower mediolateral insertion ratio indicates a more lateral entry point. (2) Sagittal plane analysis: The shortest distance (r) between the tibial plateau surface and the intramedullary nail was measured along a perpendicular line drawn from the plateau (Figure 1C). This distance indicates the proximal–distal relationship of the insertion site.

Two orthopedic surgeons who were blinded to the surgical approach independently performed all measurements. The average of the two observers’ measurements was used for the analyses. A two-way random-effects intraclass correlation coefficient (ICC) was used to assess interobserver reliability for absolute agreement based on the mean of two observers (ICC(2, k)), with 95% confidence intervals.

### 2.7. Outcomes

The primary outcome includes the difference in the nail insertion point among the three approaches in both the coronal and sagittal planes. The secondary outcome involves a pairwise comparison of the insertion points between the two approaches.

### 2.8. Statistical Analysis

Continuous variables are presented as mean (range) or median [interquartile range], as appropriate. Categorical variables were compared using the chi-square test or Fisher’s exact test, as appropriate. The Shapiro–Wilk test was used to assess the normality of the data distribution. As the data were nonnormally distributed, the Kruskal–Wallis test was conducted to compare the three groups. Post hoc pairwise comparisons were conducted using Dunn’s multiple comparison tests. A *p*-value of <0.05 indicated statistical significance. Prism 10 (GraphPad Software, San Diego, CA, USA) was used for all analyses.

## 3. Results

### 3.1. Patient Characteristics

This study included 68 patients (23 females, 45 males; mean age, 55.9 years; range, 16–104 years). The right and left tibia were affected in 40 and 28 cases, respectively. According to the AO/OTA classification, 41 (60.3%), 26 (38.2%), and 1 (1.5%) fractures were types 42A, 42B, and 42C, respectively. Fracture location was categorized as proximal 5 (7.4%), middle 27 (39.7%), and distal 36 (52.9%). An open fracture was present in 27 cases (39.7%). The distribution of surgical approaches was LPA, IPA, and SPA in 31 (45.6%), 27 (39.7%), and 10 (14.7%) patients, respectively. The mean operative time was 119 min (range, 69–215 min). Patient and fracture characteristics, including age, sex, side, fracture type, fracture location, and open fracture status, did not significantly differ among the three groups (Table 1). No intraoperative complications related to the insertion approach were observed.

### 3.2. Coronal Plane Analysis

The median insertion ratio (b/a × 100%), representing the mediolateral position of the nail entry point, significantly differed among the three approaches (Kruskal–Wallis test, *p* < 0.0001). Interobserver agreement was good for the coronal mediolateral insertion ratio (ICC(2, k) = 0.886, 95% CI 0.82–0.93).

**Figure 2:**
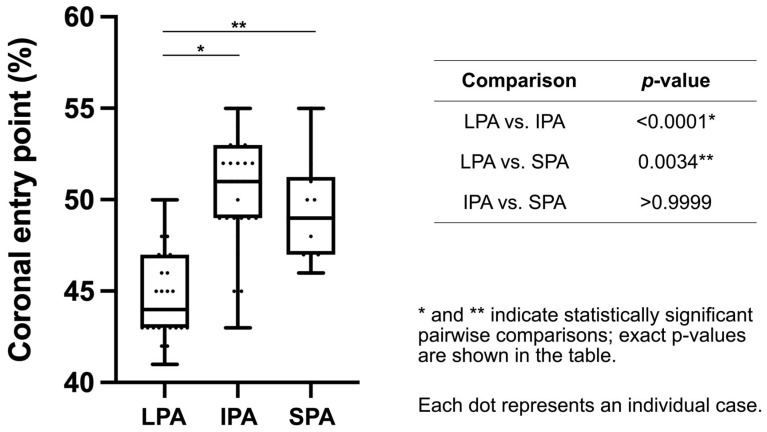
Pairwise comparison of mediolateral nail entry point ratios in the coronal plane among the three approaches. Data are presented as box-and-whisker plots, indicating the median and interquartile range. The whiskers represent the minimum and maximum values. *p*-values were based on Dunn’s multiple comparisons test following the Kruskal–Wallis test. LPA, lateral parapatellar approach; IPA, infrapatellar approach; SPA, suprapatellar approach.

Dunn’s post hoc analysis revealed that the LPA group had a significantly more lateral entry point than the IPA (*p* < 0.0001) and SPA groups (*p* = 0.0034), whereas no significant difference was observed between the IPA and SPA groups (*p* > 0.9999). The median insertion ratios were 44.0% [43.0–47.0], 51.0% [49.0–53.0], and 49.0% [47.0–51.3] in the LPA, IPA, and SPA groups, respectively. These results indicate that nails inserted via the LPA tend to start from a more lateral entry point on the tibial plateau than those inserted via other approaches (Table 2, Figure 2).

### 3.3. Sagittal Plane Analysis

The shortest distance (*r*) from the tibial articular surface to the intramedullary nail, representing the proximal–distal position of the insertion, also significantly differed among the three approaches (Kruskal–Wallis test, *p* < 0.0001). Interobserver agreement was excellent for sagittal distance r (ICC(2, k) = 0.970, 95% CI 0.95–0.98).

**Figure 3:**
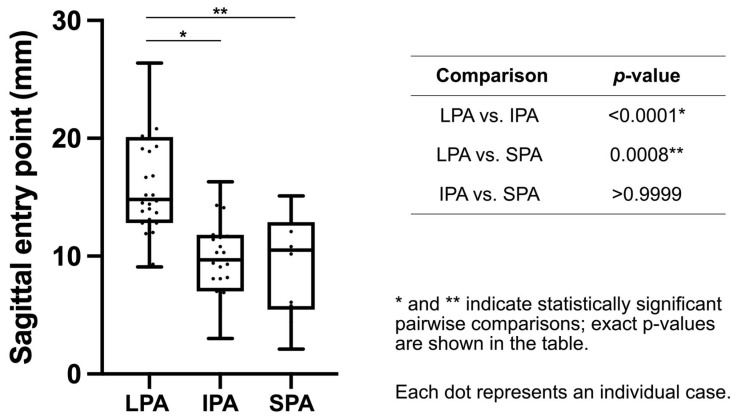
Pairwise comparison of proximal–distal nail entry point distances in the sagittal plane among the three approaches. Data are presented as box-and-whisker plots, indicating the median and interquartile range. The whiskers represent the minimum and maximum values. *p*-values were based on Dunn’s multiple comparisons test following the Kruskal–Wallis test. LPA, lateral parapatellar approach; IPA, infrapatellar approach; SPA, suprapatellar approach.

The median distances were 14.8 mm [12.8–20.1] (range, 9.1–26.4 mm), 9.7 mm [7.0–11.8] (range, 3.0–16.3 mm), and 10.5 mm [5.5–12.9] (range, 2.1–15.1 mm) in the LPA, IPA, and SPA groups, respectively. Dunn’s post hoc analysis revealed that the LPA group demonstrated a significantly more distal insertion than the IPA (*p* < 0.0001) and SPA groups (*p* = 0.0008). No significant difference was observed between the IPA and SPA groups (*p* > 0.9999) (Table 2, Figure 3). These findings indicate that the LPA tends to produce a more distal entry point relative to the tibial plateau than the IPA or SPA.

## 4. Discussion

This retrospective 3D-CT analysis compared the nail entry points associated with the LPA, IPA, and SPA for tibial shaft fracture nailing. The main finding was that the LPA led to significantly more lateral and distal insertion points than the IPA and SPA, whereas no statistically significant differences were detected between the IPA and SPA in either the coronal or sagittal plane. These results indicate that the LPA provides a less invasive extra-articular route; however, it may carry a higher risk of suboptimal entry alignment, particularly for fractures that involve the proximal metaphysis.

### 4.1. Coronal Plane: Lateral Deviation and Coronal Alignment

Our coronal plane analysis revealed that the LPA produced a significantly more lateral entry point on the tibial plateau than the IPA and SPA, whereas no statistically significant difference in mediolateral position was observed between the latter two approaches. A lateralized entry point has been associated with valgus deformity in proximal tibial fractures because the nail tends to follow the medullary canal toward the center of the diaphysis, whereas the proximal fragment is displaced laterally [6,7,21,22,23]. Deviation toward the lateral plateau edge increases the risk of coronal malalignment, particularly in metaphyseal fractures [1,6,7,8,22,23].

In the present study, both the IPA and SPA permitted the guidewire to be placed close to the central corridor, whereas the LPA tended to shift the entry further laterally. With the LPA, the patellar tendon and surrounding soft-tissue envelope limit the medial direction of the instruments; thus, surgeons often perceive that they are aiming centrally, although the final entry point is relatively lateral. Several clinical studies have reported acceptable overall alignment after tibial nailing using LPA or extra-articular semiextended techniques [2,12,15,16,17]. However, these studies primarily relied on 2D radiographs and did not directly quantify the mediolateral position of the entry point. Our 3D-CT analysis adds to the literature by revealing that, even when postoperative radiographs appear satisfactory, the LPA systematically yields a more lateral entry position than the IPA and SPA and may place the entry point outside the recommended central corridor if no conscious effort is made to direct the guidewire medially.

Altogether, these findings suggest the practice of intentionally aiming the guidewire slightly medially under true anteroposterior fluoroscopic views when using the LPA to land within the central safe zone of the plateau and prevent excessive lateral deviation. In clinical practice, these geometric characteristics are particularly crucial in fractures that involve the proximal metaphysis or proximal diaphysis, where even small deviations in the coronal plane translate into clinically relevant alignment issues [6,21]. Approaches for proximal fracture patterns that facilitate a central entry point in a semiextended position, such as the SPA, have been advocated and are frequently preferred because they enable easier maintenance of the mechanical axis during guidewire and nail insertion [24,25]. In contrast, the LPA may be better suited for diaphyseal or distal extra-articular fractures. The LPA selected near the proximal metaphysis may warrant careful attention, and surgeons may consciously aim the guidewire more medially and consider adjuncts, such as blocking screws, to reduce the risk of valgus deformity [25]. Because postoperative alignment was not directly assessed, the clinical implications of the observed entry-point differences should be interpreted cautiously.

### 4.2. Sagittal Plane: Distal Entry and Choice of Approach

In the sagittal plane, the LPA led to a significantly more distal insertion than the IPA and SPA, whereas the latter two approaches showed no statistically significant differences in proximal–distal positions. A distal starting point reduces the amount of proximal metaphyseal bone stock available for locking screws and may compromise fixation in fractures that extend into the proximal metaphysis. Further, the more distal the starting point, the shorter the lever arm with which the nail controls the proximal fragment, thereby potentially increasing the risk of procurvatum or other sagittal malalignment in proximal diaphyseal fractures. Previous radiographic and CT-based studies have emphasized the importance of maintaining an appropriate relationship between the nail, joint surface, and proximal locking screws to prevent loss of fixation and malalignment, particularly in the metaphyseal regions [5].

Our findings indicate that the IPA and SPA enable a relatively proximal entry point to be achieved, despite differences in patient positioning and the trajectory of the guide instruments. This observation is consistent with reports indicating that semiextended SPA nailing can reproduce a starting point similar to that of the conventional IPA while facilitating reduction and alignment control in proximal fractures [4,9,12]. These clinical studies have generally focused on postoperative alignment and functional outcomes rather than direct measurement of the entry point. However, they support the concept that a semiextended technique helps maintain the mechanical axis by keeping the tibia closer to the weight-bearing position during guidewire and nail insertion.

In contrast, the LPA in our series tended to produce a more distal entry, despite being performed in a semiextended position. Technique descriptions of extra-articular LPA nailing emphasize creating a cortical window slightly anterior and distal to the proximal tibia to remain outside the joint capsule and protect the patellofemoral cartilage [1,3,26,27]. In practice, this extra-articular trajectory, in combination with lateral access around the patellar tendon, encourages the surgeon to start a few millimeters further from the joint surface, which is consistent with the larger plateau–nail distance observed in the LPA group in our CT analysis. Recent clinical series of LPA or extra-articular semiextended nailing have reported satisfactory alignment and union rates in both shaft and metaphyseal–diaphyseal junction fractures. However, they did not quantify the proximal–distal position of the entry point [2,16,17,18]. Our 3D-CT analysis complements these studies by revealing a systematic tendency toward a more distal entry when the LPA is employed.

From a practical standpoint, these results indicate that for fractures that involve the proximal metaphysis or proximal diaphysis, surgeons should be cautious when using the LPA and consciously aim for the most proximal acceptable starting point while maintaining a perpendicular relationship to the joint surface on true lateral fluoroscopic views. In such cases, an SPA semiextended technique may provide advantages in terms of alignment control because it combines a central entry position with easier maintenance of the mechanical axis, whereas alignment control with the IPA in knee flexion is more challenging. The LPA may still provide the benefits of extra-articular access and potentially reduced anterior knee pain, as reported in previous clinical studies, for more diaphyseal or distal fractures, where control of the proximal fragment is less critical and a slightly more distal entry may be acceptable.

### 4.3. Clinical Implications

The present study has several practical implications for daily practice. First, no statistically significant differences in the entry point positions were observed between the IPA and SPA groups in this cohort. The mediolateral insertion ratio (b/a × 100%) in the coronal plane provides a size-normalized measure that facilitates comparison across patients with different tibial plateau widths. Positioning in the sagittal plane was quantified using the absolute distance r (mm). This finding may reassure surgeons who are concerned that the semiextended SPA may alter the ideal starting point. This is consistent with previous clinical and meta-analytic studies reporting that SPA nailing yields alignment and union rates similar to or better than those of the IPA, while potentially facilitating reduction and nail insertion in a semiextended position [4,9,12]. Thus, the selection between IPA and SPA for tibial shaft and proximal diaphyseal fractures can reasonably be based on the surgeon’s experience, available instrumentation, and the relative importance of anterior knee pain versus ease of alignment control.

Second, the tendency of the LPA to produce lateral and distal entry points emphasizes the need for careful attention to guidewire placement when using this technique, as previously reported. Several clinical series have indicated that LPA or extra-articular semiextended approaches achieve satisfactory alignment, union, and functional outcomes, and may even reduce anterior knee pain compared with traditional IPA nailing [10,15,16,17,18,28]. However, these studies have largely relied on 2D radiographs and clinical scores without directly quantifying the 3D entry point. Our CT-based findings indicate that, despite acceptable overall alignment on radiographs, the LPA systematically yields a more lateral and distal starting point than the IPA or SPA. Surgeons using the LPA should be particularly vigilant regarding coronal and sagittal alignments in fractures that involve the proximal metaphysis or proximal diaphysis, where small deviations in the entry position can translate into valgus or procurvatum deformities. In practice, this may include consciously aiming the guidewire more medially and proximally under true anteroposterior and lateral fluoroscopic views and considering adjuncts, including blocking screws and semiextended positioning, when treating proximal fracture patterns.

Third, to help clinical interpretation, our mediolateral insertion ratio can be viewed in the context of previously described “target corridors” for tibial nail entry points [1]. Previous anatomic and radiographic studies have recommended a relatively central starting point within the proximal tibial plateau to reduce the risk of malalignment, particularly in proximal fracture patterns [29,30]. In the present study, we did not impose a single strict corridor threshold or calculate out-of-corridor rates because such corridors differ across implant systems and imaging definitions; however, our data consistently indicate that the LPA generates a more lateral starting point than the IPA or SPA. Therefore, when using the LPA, surgeons should be mindful of maintaining the entry point within an appropriate medial–lateral corridor under standard true anteroposterior and lateral fluoroscopic views, especially for fractures that involve the proximal diaphysis/metaphysis.

Finally, our results may help refine the approach selection according to the fracture location. A slightly more distal entry may be acceptable for more diaphyseal or distal extra-articular fractures, because control of the proximal fragment is less critical, and the LPA may provide the advantages of extra-articular access, easier positioning, and potentially less anterior knee pain, as reported in previous studies. In contrast, for fractures extending into the proximal metaphysis or proximal diaphysis, our data support the use of approaches that facilitate a central and relatively proximal entry point, including the semiextended SPA or carefully performed IPA nailing, while reserving the LPA for cases in which its benefits clearly outweigh the potential risk of suboptimal entry alignment. Therefore, incorporating 3D information about entry point geometry into preoperative planning may improve decision-making and help optimize both alignment and functional outcomes in tibial intramedullary nailing.

### 4.4. Strengths and Limitations

This study had several strengths. We used postoperative 3D-CT reconstructions to standardize the evaluation of the nail entry point, which is more accurate than simple 2D radiographs. All cases were treated with reamed and locked tibial nails with similar proximal geometries, which minimize the effect of the implant design on the entry point. Further, all surgeries were performed at a single institution, which reduced the variability in the surgical technique and postoperative imaging.

However, this study has limitations to be acknowledged. First, this retrospective, single-center study comprised an imbalanced sample size across approach groups, particularly the SPA group, and approach selection was at the surgeon’s discretion. Therefore, selection bias, temporal (learning-curve) effects, and residual confounding cannot be excluded. Second, we focused on entry-point geometry and did not evaluate postoperative alignment or other clinical outcomes (e.g., union or anterior knee pain). Thus, the clinical implications of the observed differences should be interpreted as hypothesis-generating. Third, our CT-based evaluation quantified the entry point only in the coronal and sagittal planes, and the cohort was restricted to patients with available postoperative CT. Further, multiple nail systems were employed, and a small implant-specific influence cannot be fully excluded. These factors may limit generalizability.

### 4.5. Future Directions

Despite these limitations, our results provide useful information regarding the geometric characteristics of the nail entry point related to the three commonly employed approaches for tibial nailing. Future prospective studies with larger cohorts are warranted to confirm the present findings and investigate the association between entry point deviation and clinical outcomes, particularly in proximal tibial fractures.

## 5. Conclusions

This retrospective 3D-CT study revealed that the LPA for tibial shaft fracture nailing was associated with a significantly more lateral and distal nail entry point than the IPA and SPA, whereas no statistically significant difference was detected between the IPA and SPA groups. Surgeons should consider that the LPA may be associated with coronal malalignment, particularly when fractures extend into the proximal diaphysis, and should pay careful attention to guidewire placement and alignment. For such fractures, the SPA in a semiextended position may facilitate achieving the intended entry point and mechanical axis compared with the conventional IPA with knee flexion.

## Figures and Tables

**Table 1:** Patient and Surgical Characteristics.

Variable	All (n = 68)	LPA (n = 31)	IPA (n = 27)	SPA (n = 10)	*p*-Value
Age (years)	55.9 (16–104)	53.9 (18–91)	56.1 (16–104)	58.9 (20–85)	0.827
Sex Female Male	23 45	8 23	9 18	6 4	0.143
Side Right Left	40 28	12 19	11 16	5 5	0.845
Fracture type 42A 42B 42C	41 26 1	19 12 0	18 9 0	4 5 1	0.218
Fracture location proximal middle distal	5 27 36	2 15 14	1 8 18	2 4 4	0.237
Open fracture	27 (39.7%)	14 (45.2%)	9 (33.3%)	4 (40.0%)	0.629
Operative time (min)	119 (69–215)	121 (75–184)	121 (69–215)	106 (75–155)	0.267

Abbreviations: LPA, lateral parapatellar approach; IPA, infrapatellar approach; SPA, suprapatellar approach. Values are expressed as mean (range) or number of patients. The Kruskal–Wallis test was used to calculate *p*-values for continuous variables. The Chi-square test or Fisher’s exact test was used to calculate *p*-values for categorical variables, as appropriate.

**Table 2:** Comparison of coronal and sagittal entry point measurements among the three approaches.

Parameter	LPA (n = 31)	IPA (n = 27)	SPA (n = 10)	*p*-Value
Coronal (%)	44.0 [43.0–47.0]	51.0 [49.0–53.0]	49.0 [47.0–51.3]	<0.0001
Sagittal (mm)	14.8 [12.8–20.1]	9.7 [7.0–11.8]	10.5 [5.5–12.9]	<0.0001

Abbreviations: LPA, lateral parapatellar approach; IPA, infrapatellar approach; SPA, suprapatellar approach. Values are expressed as median [interquartile range]. The Kruskal–Wallis test was used to calculate *p*-values.

## Data Availability

Due to ethical considerations, we are unable to make the full dataset publicly available. However, we are open to discussing requests for anonymized data from qualified researchers under appropriate agreements.

## Supplementary Materials

The following supporting information can be downloaded at: https://www.mdpi.com/article/10.3390/life16010087/s1, Table S1. Distribution of surgical approaches over the study period. Table S2. Distribution of intramedullary nail systems by surgical approach. Table S3. Pairwise comparison between infrapatellar (IPA) and suprapatellar (SPA) approaches using the Mann–Whitney test.

## Abbreviations

The following abbreviations are used in this manuscript:

2D	Two-dimensional
3D	Three-dimensional
AO/OTA	Arbeitsgemeinschaft für Osteosynthesefragen/Orthopaedic Trauma Association
ICC	Intraclass correlation coefficient
IPA	Infrapatellar approach
IRB	Institutional review board
LPA	Lateral parapatellar approach
SPA	Suprapatellar approach

## References

[1] Patel A.H., Wilder J.H., Lee O.C., Ross A.J., Vemulapalli K.C., Gladden P.B., Martin M.P., Sherman W.F. (2022). A Review of Proximal Tibia Entry Points for Intramedullary Nailing and Validation of The Lateral Parapatellar Approach as Extra-articular. Orthop. Rev..

[2] Baker H.P., Strelzow J., Dillman D. (2022). Tibial alignment following intramedullary nailing via three approaches. Eur. J. Orthop. Surg. Traumatol..

[3] Kubiak E.N., Widmer B.J., Horwitz D.S. (2010). Extra-articular technique for semiextended tibial nailing. J. Orthop. Trauma..

[4] Lu Y., Wang G., Hu B., Ren C., Sun L., Wang Z., He C., Xue H., Li Z., Zhang K. (2020). Comparison of suprapatellar versus infrapatellar approaches of intramedullary nailing for distal tibia fractures. J. Orthop. Surg. Res..

[5] Schumaier A.P., Avilucea F.R., Southam B.R., Sinha P., Le T.T., Wyrick J.D., Archdeacon M.T. (2020). Terminal position of a tibial intramedullary nail: A computed tomography (CT) based study. Eur. J. Trauma. Emerg. Surg..

[6] Freedman E.L., Johnson E.E. (1995). Radiographic analysis of tibial fracture malalignment following intramedullary nailing. Clin. Orthop. Relat. Res..

[7] Maslow J.I., Joseph H.L., Hong D.Y., Henry A.L., Mitchell P.M., Collinge C.A. (2020). Radiographic Evaluation of the Tibial Intramedullary Nail Entry Point. J. Am. Acad. Orthop. Surg..

[8] McConnell T., Tornetta P., Tilzey J., Casey D. (2001). Tibial portal placement: The radiographic correlate of the anatomic safe zone. J. Orthop. Trauma..

[9] Ponugoti N., Rudran B., Selim A., Nahas S., Magill H. (2021). Infrapatellar versus suprapatellar approach for intramedullary nailing of the tibia: A systematic review and meta-analysis. J. Orthop. Surg. Res..

[10] Bakhsh W.R., Cherney S.M., McAndrew C.M., Ricci W.M., Gardner M.J. (2016). Surgical approaches to intramedullary nailing of the tibia: Comparative analysis of knee pain and functional outcomes. Injury.

[11] Ozcan C., Turkmen I., Sokucu S. (2020). Comparison of three different approaches for anterior knee pain after tibia intramedullary nailing. Eur. J. Trauma. Emerg. Surg..

[12] Wang C., Chen E., Ye C., Pan Z. (2018). Suprapatellar versus infrapatellar approach for tibia intramedullary nailing: A meta-analysis. Int. J. Surg..

[13] Ladurner A., Acklin Y.P., Mueller T.S., Sommer C. (2019). Decrease surgery time by using an alternative lateral parapatellar approach for tibia shaft fracture nailing. Arch. Orthop. Trauma. Surg..

[14] Zamora R., Wright C., Short A., Seligson D. (2016). Comparison between suprapatellar and parapatellar approaches for intramedullary nailing of the tibia. Cadaveric study. Injury.

[15] Alessio-Mazzola M., Alpi V., Ghezzi E., Placella G., Salini V. (2025). A Retrospective Study With 2-Year Follow-up Comparing Semi-Extended Tibia Nailing Techniques: The Suprapatellar Versus the Extra-Articular Lateral Parapatellar Approach. HSS J..

[16] Cao X., Tang Q., Zhou B., Xiao W., Chen H. (2024). Comparison of the efficacy of intramedullary nailing via the lateral parapatellar approach versus the infrapatellar approach in the treatment of tibial metaphyseal-diaphyseal junction fractures. J. Orthop. Surg. Res..

[17] Nie W., Wang Z., Xu S., Guo S., Yue Y., Sun K. (2024). A retrospective investigation on clinical and radiographic outcomes of distal tibial fractures after intramedullary nailing using the lateral parapatellar extra-articular approach. Arch. Orthop. Trauma. Surg..

[18] Yang L., Nie G. (2025). Comparison of lateral parapatellar vs. infrapatellar approaches for intramedullary nailing for tibial shaft fractures. Front. Surg..

[19] Kanezaki S., Miyazaki M., Ishida T., Hino A., Kawagishi M., Sakamoto T., Kaku N. (2025). The anterior offset of the standard entry point for tibial intramedullary nails: A transparent 3D-CT image based analysis. J. Orthop..

[20] Meinberg E.G., Agel J., Roberts C.S., Karam M.D., Kellam J.F. (2018). Fracture and Dislocation Classification Compendium-2018. J. Orthop. Trauma.

[21] Lang G.J., Cohen B.E., Bosse M.J., Kellam J.F. (1995). Proximal third tibial shaft fractures. Should they be nailed?. Clin. Orthop. Relat. Res..

[22] Weninger P., Tschabitscher M., Traxler H., Pfafl V., Hertz H. (2010). Intramedullary nailing of proximal tibia fractures--an anatomical study comparing three lateral starting points for nail insertion. Injury.

[23] Stinner D.J., Mir H. (2014). Techniques for intramedullary nailing of proximal tibia fractures. Orthop. Clin. N. Am..

[24] Cicekli O., Topcu H.N., Kochai A., Sukur E., Turker M. (2019). Comparison of suprapatellar and infrapatellar tibial nailing: More anatomic entry point and fracture reduction via the suprapatellar approach. Int. J. Clin. Exp. Med..

[25] Wu D.H., Zhang Y., Xu D., Lou W.G., Zhang J.Y. (2025). Evaluation of the effectiveness of suprapatellar versus infrapatellar approach in intramedullary nailing for the treatment of tibial fractures. Eur. J. Med. Res..

[26] Ghezzi E., Alpi V., Alessio-Mazzola M., Placella G., Salini V. (2022). Surgical technique for lateral extra-articular parapatellar approach for tibia nailing: A technical note. J. Orthop..

[27] Stella M., Santolini E., Felli L., Santolini F., Horwitz D.S. (2019). Semiextended Tibial Nail Insertion Using an Extraarticular Lateral Parapatellar Approach: A 24-Month Follow-up Prospective Cohort Study. J. Orthop. Trauma..

[28] Mishra J., Pani S., Das T., Khandelwal C., Mishra S. (2024). The Lateral Para-Patellar Approach for Intramedullary Tibia Nailing in Distal Tibia Extra-articular Fractures: A Prospective Cohort Study. Cureus.

[29] Althausen P.L., Neiman R., Finkemeier C.G., Olson S.A. (2002). Incision placement for intramedullary tibial nailing: An anatomic study. J. Orthop. Trauma.

[30] Yasuda T., Sato K., Yamazaki K., Arai M., Shinohara D., Taisuke Y., Minagawa Y., Samejima Y., Okamoto K., Irie Y. (2022). Nail insertion points in semi-extended nailing of tibial fractures and their influence on alignment: A retrospective cohort study comparing two nail insertion techniques. Injury.

