# Supplementary material for: Comparison of Tibial Nail Entry Point Location Among Infrapatellar, Suprapatellar, and Lateral Parapatellar Approaches Using Postoperative 3D-CT
Source: Life (Basel). 2026 Jan 7;16(1):87. doi: 10.3390/life16010087 (PMC12843317; doi:10.3390/life16010087)
Supplement: Supplementary file 1 [file life-16-00087-s001.zip › Table S1.pdf]

Table S1. Distribution of surgical approaches over the study period

Period	LPA (n = 31)	IPA (n = 27)	SPA (n = 10)	Total (n = 68)
Early (2014–2018)	1 (3.8%)	22 (84.6%)	3 (11.5%)	26 (38.2%)
Late (2019–2024)	30 (71.4%)	5 (11.9%)	7 (16.7%)	42 (61.8%)

Values are shown as n (within-period %). P-values were calculated using Fisher's exact test ($p < 0.001$).
