# Supplementary material for: Comparison of Tibial Nail Entry Point Location Among Infrapatellar, Suprapatellar, and Lateral Parapatellar Approaches Using Postoperative 3D-CT
Source: Life (Basel). 2026 Jan 7;16(1):87. doi: 10.3390/life16010087 (PMC12843317; doi:10.3390/life16010087)
Supplement: Supplementary file 1 [file life-16-00087-s001.zip › Table S2.pdf]

Table S2. Distribution of intramedullary nail systems by surgical approach

Implant system (manufacturer)	All (n = 68)	LPA (n = 31)	IPA (n = 27)	SPA (n = 10)
Expert Tibial Nail / Tibial Nailing System（Synthes）	19 (27.9%)	7 (22.6%)	10 (37.0%)	2 (20.0%)
T2 Tibial Nail（Stryker）	22 (32.4%)	11 (35.5%)	5 (18.5%)	6 (60.0%)
Natural Nail Tibial System（Zimmer Biomet）	22 (32.4%)	10 (32.3%)	11 (40.7%)	1 (10.0%)
Phoenix Tibial Nail（Zimmer Biomet）	1 (1.5%)	0	1 (3.7%)	0
TRIGEN Meta-Nail（S&N）	4 (5.9%)	2 (6.5%)	1 (3.7%)	1 (10.0%)

Data are presented as n (%). The overall distribution of implant systems across approaches was compared using Fisher's exact test (p = 0.216). Due to small cell counts in several categories, we did not perform implant-adjusted multivariable analyses; instead, we report the implant distribution for transparency.
