# Supplementary material for: Comparison of Tibial Nail Entry Point Location Among Infrapatellar, Suprapatellar, and Lateral Parapatellar Approaches Using Postoperative 3D-CT
Source: Life (Basel). 2026 Jan 7;16(1):87. doi: 10.3390/life16010087 (PMC12843317; doi:10.3390/life16010087)
Supplement: Supplementary file 1 [file life-16-00087-s001.zip › Table S3.pdf]

Table S3. Pairwise comparison between infra-patellar (IPA) and supra-patellar (SPA) approaches using the Mann–Whitney test (Hodges–Lehmann median difference with confidence interval).

Outcome	Group (n)	Median	Hodges–Lehmann median difference (SPA–IPA)	95% CI	Mann–Whitney U	*p*-value
Coronal position (%)	IPA (27)<br>SPA (10)	51.0<br>49.0	−2.0 pp	−4.0 to 1.0	97.5	0.203
Sagittal offset (mm)	IPA (27)<br>SPA (10)	9.7<br>10.5	+0.65 mm	−2.8 to 3.8	126.5	0.781

Values are presented as medians. The Hodges–Lehmann estimator is reported as the median difference (SPA–IPA). For sagittal offset, GraphPad Prism reports the Hodges–Lehmann estimate as (IPA–SPA); therefore, the sign was reversed to present (SPA–IPA) consistently across outcomes. For coronal position, results are expressed in percentage points (pp) (ratio × 100). Two-tailed exact p values are shown.
